# Supplementary material for: Novel Tunable Spatio-Temporal Patterns From a Simple Genetic Oscillator Circuit
Source: Front Bioeng Biotechnol. 2020 Aug 28;8:893. doi: 10.3389/fbioe.2020.00893 (PMC7509427; doi:10.3389/fbioe.2020.00893)
Supplement: Supplementary file 9 [file Data_Sheet_1.PDF]

frontiers

Supplementary Material

1 PARAMETER ESTIMATES

- Cell volume (V) varies with growth rate, for a cell with $1\mu m$ height, $1\mu m$ width and $2\mu m$ length the cell volume is $2\mu m^3$, we consider a cell volume of $1\mu m^3$ or 10^{-15} litres for simplicity which is in the same order of magnitude (Churchward et al., 1981)
- Constitutive transcription (a), 0.5 transcripts per second (Elowitz and Leibler, 2000). Which is in the range of 0.1 to 1 transcripts per second (Liang et al., 1999) We considered 1 transcript as a particle so 0.5 particles are 8.3×10^{-25} moles, divided by cell volume to obtain 8.3×10^{-10}
- Leaky transcription rate (b), 5×10^{-4} transcripts per second (Elowitz and Leibler, 2000). We considered 1 transcript as a particle so 5×10^{-4} are 8.30×10^{-28} moles, divided by cell volume to obtain a concentration rate of 8.30×10^{-13} Molar/second.
- The average length of repressors LacI, TetR and λ cl is 871 bp (Elowitz and Leibler, 2000), 290.3 codons or amino acids when translated, similar to dsRed monomer with 220 amino acids. Translation rate (c), 6.7 DsRed monomers per transcript per minute (Guet et al., 2008). We consider proteins and transcripts as particles, we get particle over particle per 60s, particles get cancelled and we get a translation rate of $1.11 \times 10^{-1}s^{-1}$.
- The switching concentration (K), 40 repressors per cell (Elowitz and Leibler, 2000) We consider proteins as a particle so 40 repressor protein are 6.64×10^{-23} moles, divided by cell volume to obtain a concentration of $6.64 \times 10^{-8} Molar$.
- Growth rate ranges from $\mu = 0.2h^{-1}$ to $1.2h^{-1}$ (Andersen and von Meyenburg, 1980). We consider $\mu = 1h^{-1}$ or $2.77 \times 10^{-4}s^{-1}$.
- mRNA half life is estimated as 5-10 minutes (Taniguchi et al., 2010) giving a degradation rate of $\delta = log(2)/5 = 0.139$ per minute, which is $2.31 \times 10^{-3}s^{-1}$.
- Protein half life is taken from (Purcell et al., 2012) as 41 minutes, giving degradation rate $log(2)/41 = 0.017$ which is 2.8×10^{-4}.
- The Young's modulus of *E. coli* cells at short time scales, which in effect relates to the effective viscosity at long time scales of growth, has been estimated between 10^5 and 10^8 Pa. Typical hydrogels and PDMS achieve stifness in the range 10^3 Pa to 10^6 MPa (Seghir and Arscott, 2015). (seghi just show range between 0.8Mpa and 10Mpa) The biophysical gamma, lets call it Γ is the ratio of cell to substrate stiffness. If we take PDMS stiffness of 10^6 Pa and cell stiffness then Γ is in the range 10^{-1} and 10^2. Taking typical values we could justify using 0.1, 1, and 10.

Parameter	Meaning	Order of magnitude
a	Constitutive transcription rate of repressor genes	$10^{-9} Molar\ s^{-1}[c1]$
b	Leaky transcription rate of repressor genes	$10^{-13} Molar\ s^{-1}[c2]$
c	Translation rate of repressor genes	$10^{-1}s^{-1}[c2, c3]$
K	Switching concentration of repressors	$10^{-7}Molar[c2]$
n	Cooperativity of repressors	10^0 [c2]
δ	Degradation rate of mRNA of repressor	$10^{-3}s^{-1}[c2]$
γ	Degradation rate of repressor	$10^{-4}s^{-1}[c2]$
μ	Growth rate of *Escherichia coli*	$10^{-4}s^{-1}[c4]$

Table S1. Parameters of the two-step model of the repressilator. [c1]= (Churchward et al., 1981), [c2]= (Elowitz and Leibler, 2000), [c3]= (Guet et al., 2008), [c4]= (Andersen and von Meyenburg, 1980).

2 ESTIMATED VALUE OF α AND $\bar{\gamma}$

Using the order of magnitude estimates in table S1 we have,

$$\alpha = \frac{ac}{\delta \mu_0 K} \approx \frac{10^{-9}10^{-1}}{10^{-3}10^{-4}10^{-7}} = 10^4 \tag{S1}$$

For $\bar{\gamma}$ we have simply $\bar{\gamma} = \gamma/\mu_0 \approx 10^{-4}/10^{-4} = 1$. Based on these estimates we chose $\alpha \in (10^2, 10^5)$ and $\bar{\gamma} \in (10^{-3}, 1)$. To construct heatmaps of wavelength and wave speed we computed solutions for 25×25 uniformly log spaced values of $\bar{\gamma}$ and α.

3 GROWTH RATE PROFILE

We simulated growing colonies using accurate individual base model, CellModeller (CM) (Rudge et al., 2012) with $\Gamma = 10$ and $\Delta t = 0.05$, to extract growth rate given the biophysical constraints. We sample colonies from 5,000 cells with steps of 5,000 until 60,000 50,000 cells. Each colony size is characterized by its radius R_{max}. In S1A, we can see the characteristic exponential decrease on growth rate in colonies of different sizes. In S1B, we can see how exponential decrease shape is conserved across the different colony sizes when compared in terms of distance from edge r.

4 STOCHASTIC SIMULATION

We simulated the model of Potvin-Trottier et al. (2016) which consists of a simple one step protein production and degradation, and plasmid replication and degradation, using the Gillespie stochastic simulation algorithm (Gillespie, 1977). The parameters of the model are K the switching point in number of repressor proteins per cell, λ the maximum protein expression rate, n the cooperativity, b the mean protein burst size, N_0 the steady state number of plasmids. Following table S1 we set parameters $K = 100$, $\lambda = 100$, $n = 2$, $b = 10$, $N_0 = 10$. We chose these parameters following Potvin-Trottier et al. (2016), and approximating the order of magnitude of $\lambda = acVN_A/\delta$ based on the parameters in table S1, with V the cell volume (10^{-15} liters) and N_A Avogadro's constant. An example simulation is shown in figure S2, showing regular sustained oscillations. We set parameters $K = 10^3$, $\lambda = 10^5$, $n = 2$, $b = 1$, $N_0 = 100$. These parameters correspond to $\alpha = 10^4$ and $\bar{\gamma} + \bar{\mu} = 1$ for our continuous model, which we showed are physiologically reasonable. We simulated 100 independent trajectories of this stochastic model showing that they sustain synchronized oscillations over at least 50 generations (figure S2).

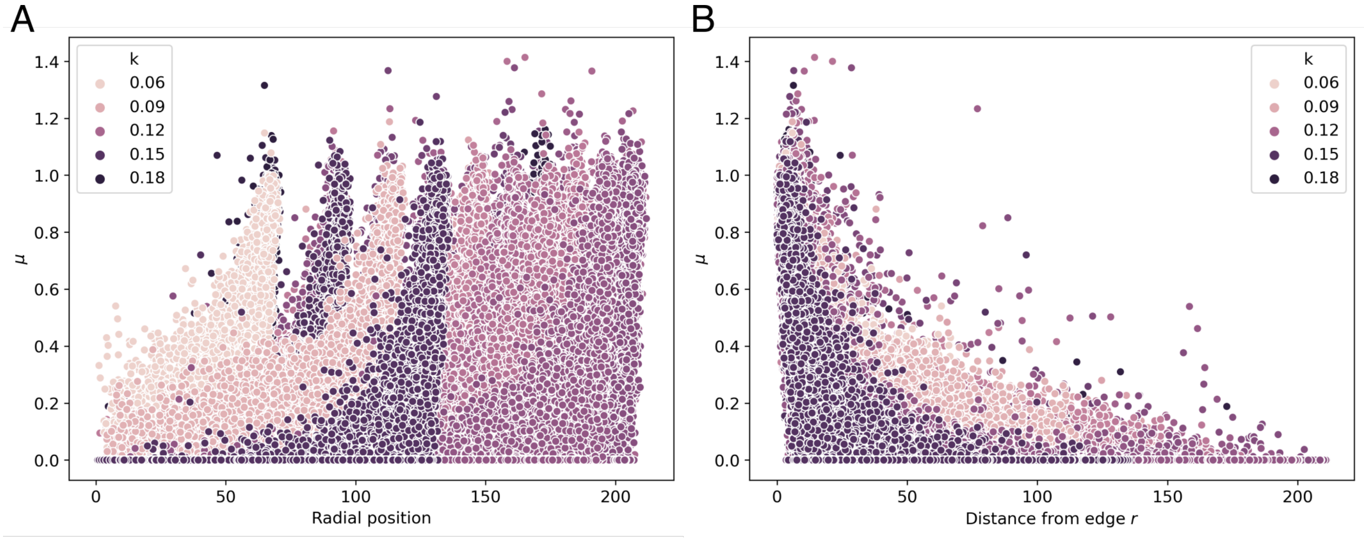

Figure S1. Growth rate profile at different colony sizes. **(A)** Distribution of individual bacteria growth rate at different R_{max} or colony sizes ordered by radial position. **(B)** Distribution of individual bacteria growth rate at different R_{max} or colony sizes ordered by distance from edge r.

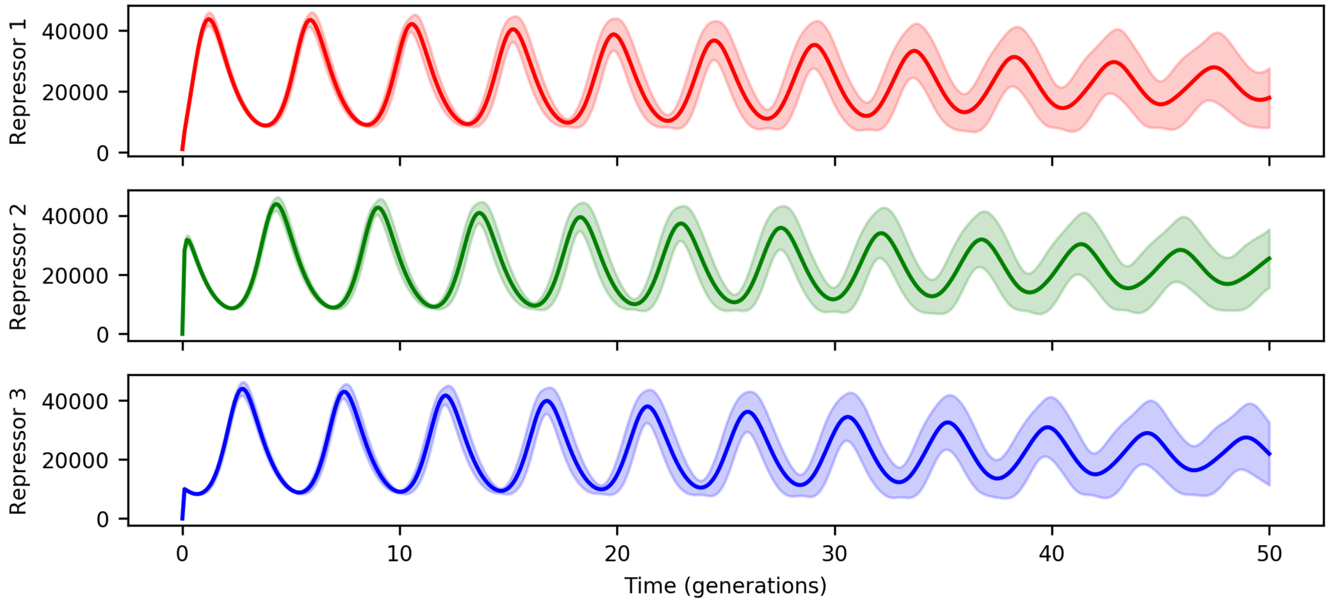

Figure S2. Stochastic simulation algorithm based on model of Potvin-Trottier et al. (2016) using $K = 10^3$, $\lambda = 10^5$, $n = 2$, $b = 1$, and $N_0 = 100$. The mean and standard deviation of 100 simulations are shown. $K = 100$, $\lambda = 100$, $n = 2$, $b = 10$, $N_0 = 10$.

5 KYMOGRAPHS OF INDIVIDUAL BASED MODEL

Colonies were grown from 1 to approximately 60,000 cells and radial averaging used to compute the kymograph, representing the spatio-temporal dynamics of the system, for a range of parameters $\bar{\gamma}$ and α (figure S3).

6 COLONIES

Here we show the four colonies analyzed in figure 5 of the main text, when composed of 60.000 50.000 cells. Figure S4 shows the colony with $\bar{\gamma}$=0.0 and α=10,000. Figure S5 shows the colony with $\bar{\gamma}$=0.3 and α=10,000. Figure S6 shows the colony with $\bar{\gamma}$=0.3 and α=100. Figure S7 shows the colony with $\bar{\gamma}$=1.0 and α=1000.

REFERENCES

Andersen, K. B. and von Meyenburg, K. (1980). Are growth rates of *Escherichia coli* in batch cultures limited by respiration? *Journal of bacteriology* 144, 114–123. doi:6998942

Churchward, G., Estiva, E., and Bremer, H. (1981). Growth rate-dependent control of chromosome replication initiation in escherichia coli. *Journal of bacteriology* 145, 1232–1238

Elowitz, M. B. and Leibler, S. (2000). A synthetic oscillatory network of transcriptional regulators. *Nature* 403, 335–338. doi:10.1038/35002125

Gillespie, D. T. (1977). Exact stochastic simulation of coupled chemical reactions. *The journal of physical chemistry* 81, 2340–2361

Guet, C. C., Bruneaux, L., Min, T. L., Siegal-Gaskins, D., Figueroa, I., Emonet, T., et al. (2008). Minimally invasive determination of mrna concentration in single living bacteria. *Nucleic acids research* 36, e73–e73

Liang, S.-T., Bipatnath, M., Xu, Y.-C., Chen, S.-L., Dennis, P., Ehrenberg, M., et al. (1999). Activities of constitutive promoters in escherichia coli. *Journal of molecular biology* 292, 19–37

Potvin-Trottier, L., Lord, N. D., Vinnicombe, G., and Paulsson, J. (2016). Synchronous long-term oscillations in a synthetic gene circuit. *Nature* 538, 514–517. doi:10.1038/nature19841

Purcell, O., Grierson, C. S., Di Bernardo, M., and Savery, N. J. (2012). Temperature dependence of ssrA-tag mediated protein degradation. *Journal of biological engineering* 6, 10. doi:10.1186/1754-1611-6-10

Rudge, T. J., Steiner, P. J., Phillips, A., and Haseloff, J. (2012). Computational Modeling of Synthetic Microbial Biofilms. *ACS Synthetic Biology* 1, 345–352. doi:10.1021/sb300031n

Seghir, R. and Arscott, S. (2015). Extended pdms stiffness range for flexible systems. *Sensors and Actuators A: Physical* 230, 33–39

Taniguchi, Y., Choi, P. J., Li, G.-W., Chen, H., Babu, M., Hearn, J., et al. (2010). Quantifying e. coli proteome and transcriptome with single-molecule sensitivity in single cells. *science* 329, 533–538

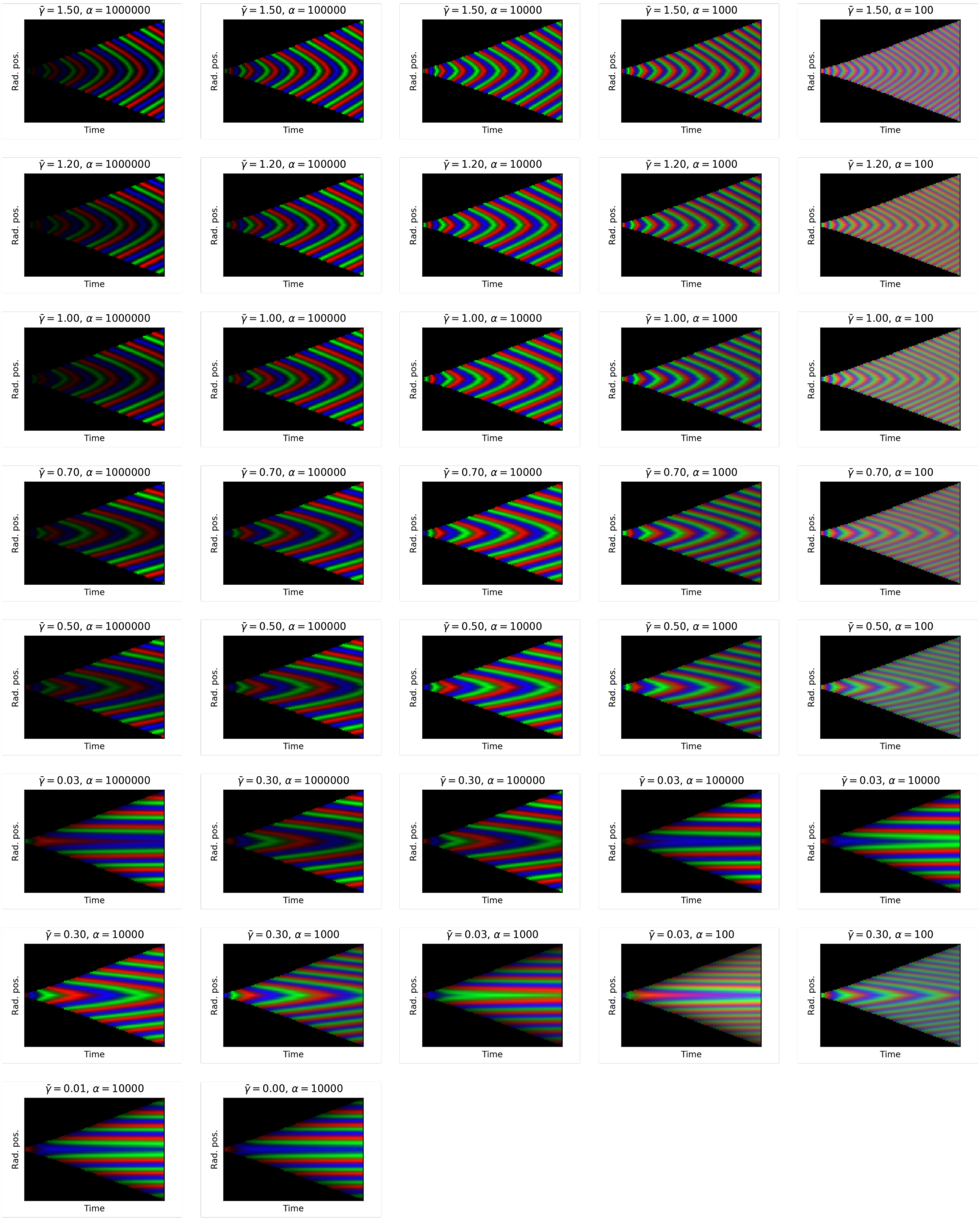

Figure S3. Kymographs computed from individual based model simulations of growing colonies from 1 to approximately 60,000 cells.

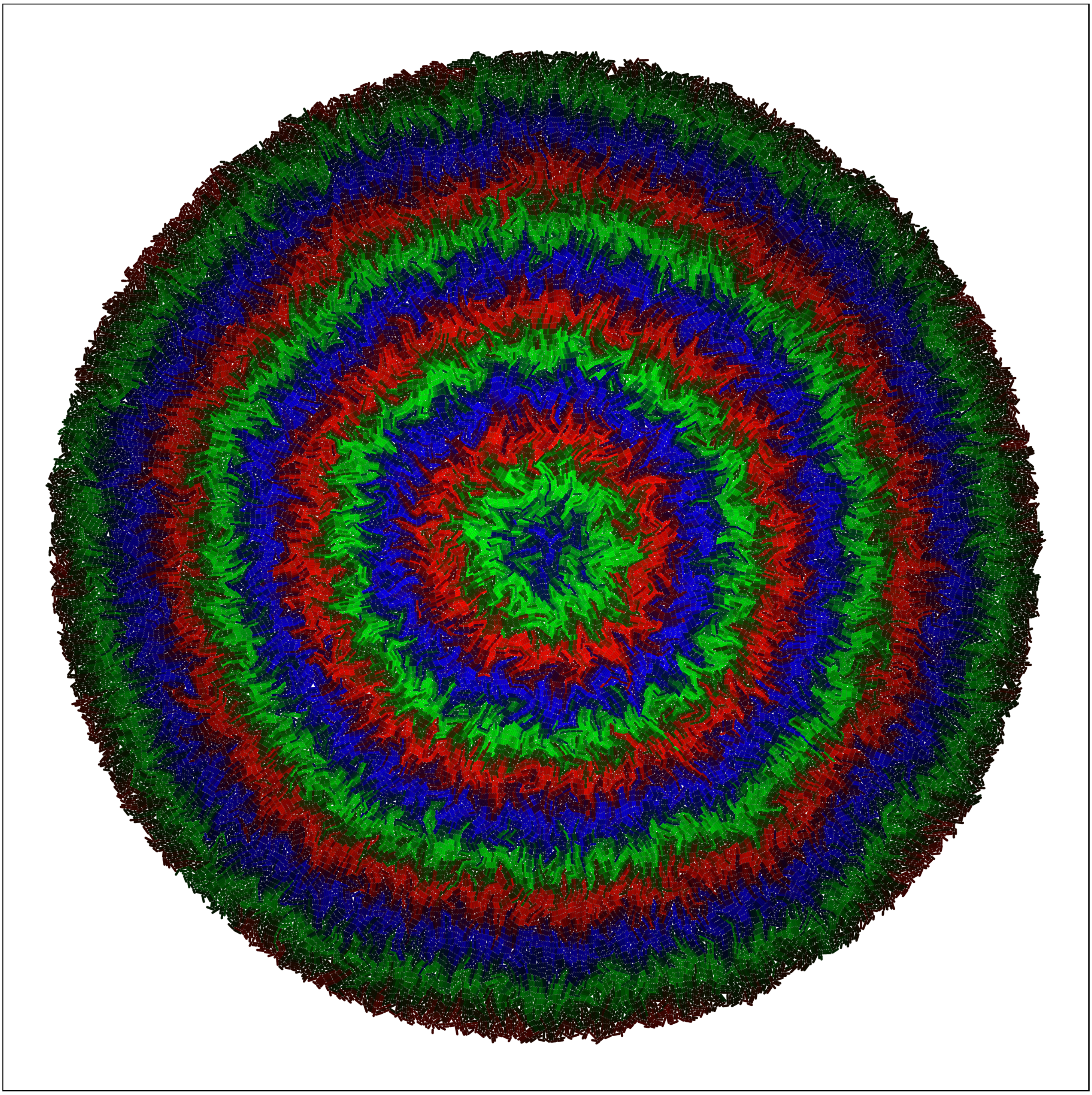

Figure S4. 60.000 50.000 cells. $\bar{\gamma}$=0 and α=10,000.

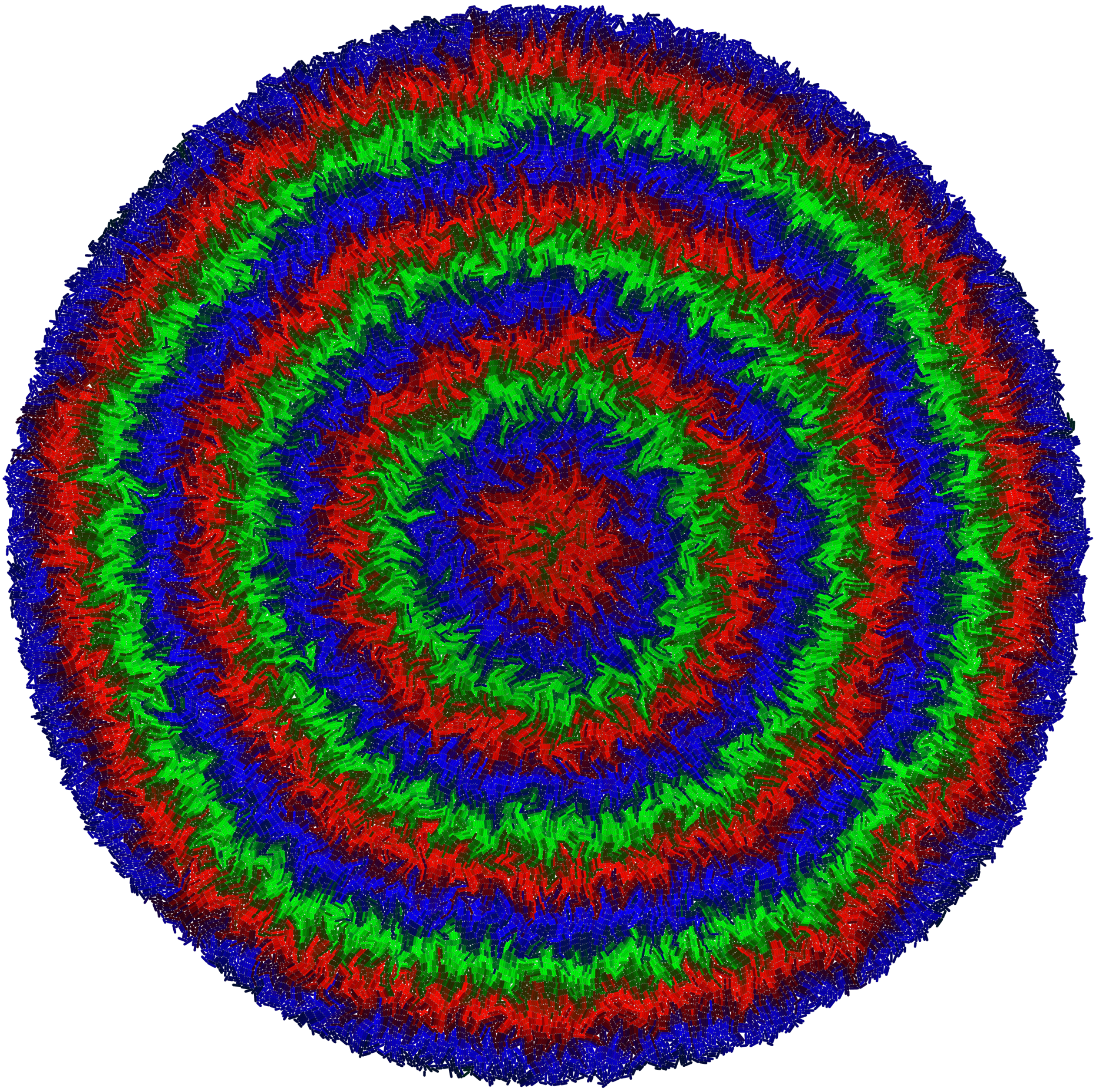

Figure S5. 60.000 50.000 cells. $\bar{\gamma}$=0.3 and α=10,000.

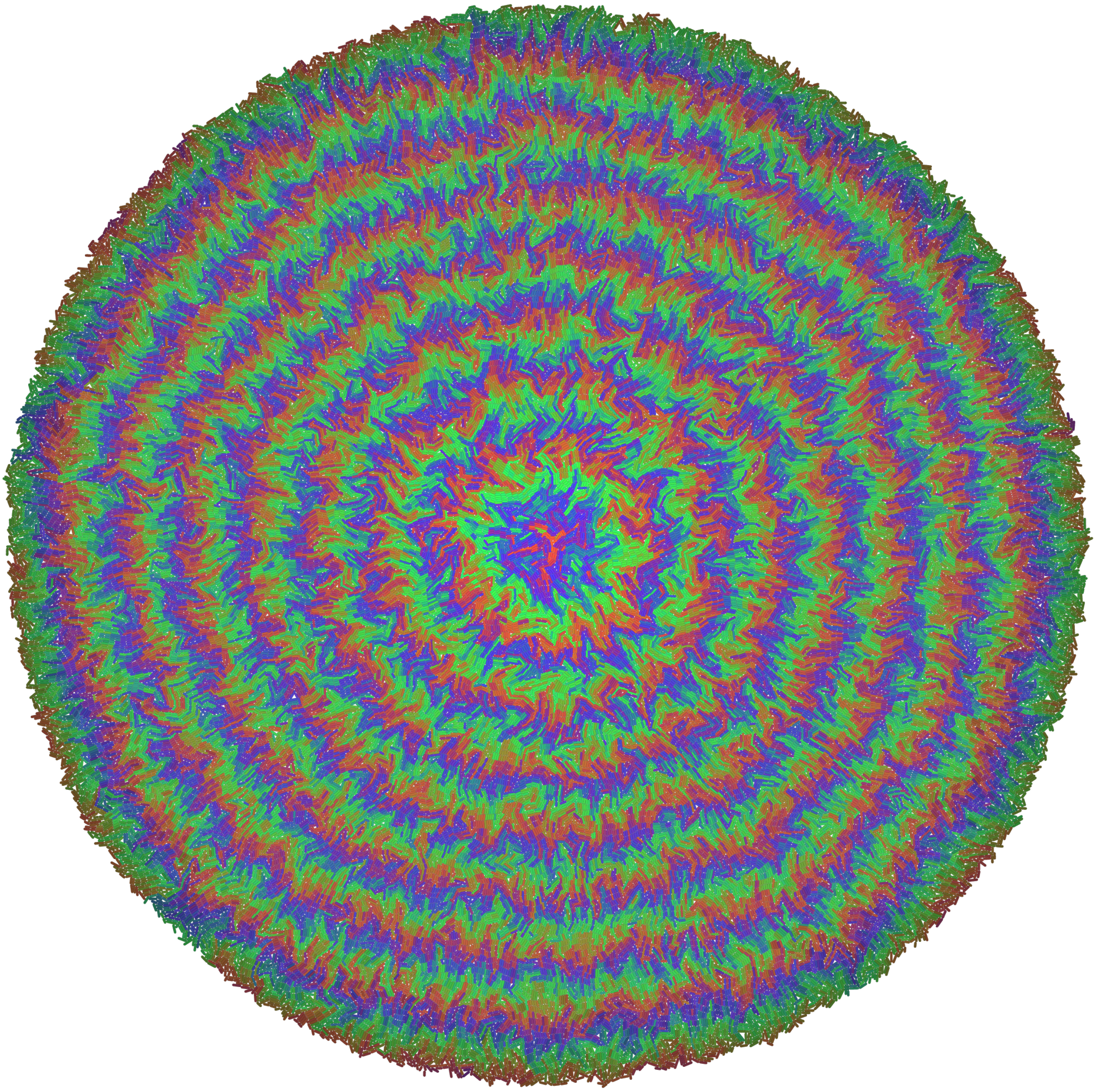

Figure S6. 60.000 50.000 cells. $\bar{\gamma}$=0.3 and α=100.

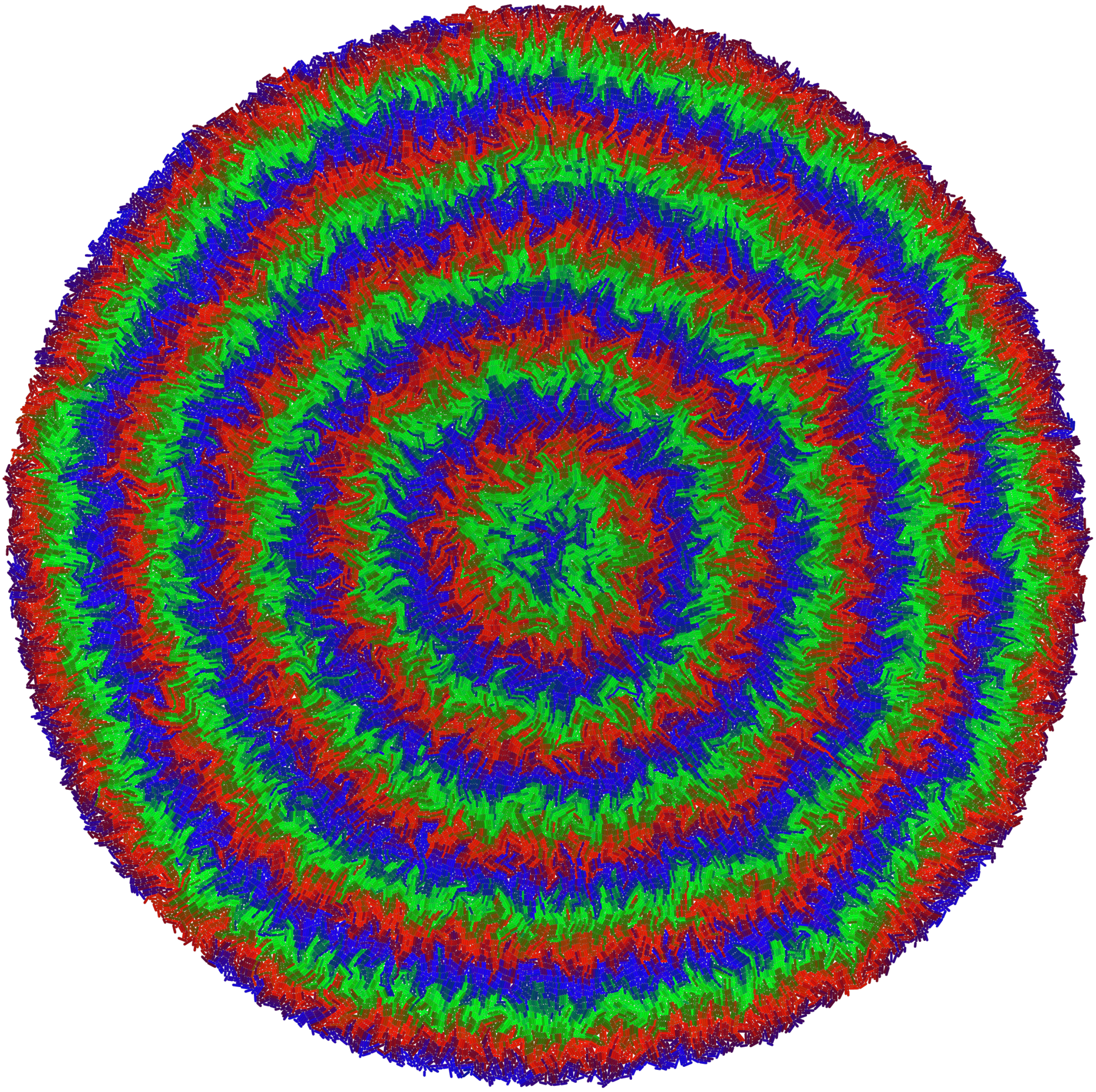

Figure S7. 60.000 50.000 cells. $\bar{\gamma}$=1 and α=1000.
