# Supplementary figures and images for: Novel Tunable Spatio-Temporal Patterns From a Simple Genetic Oscillator Circuit
Source: Front Bioeng Biotechnol. 2020 Aug 28;8:893. doi: 10.3389/fbioe.2020.00893 (PMC7509427; doi:10.3389/fbioe.2020.00893)

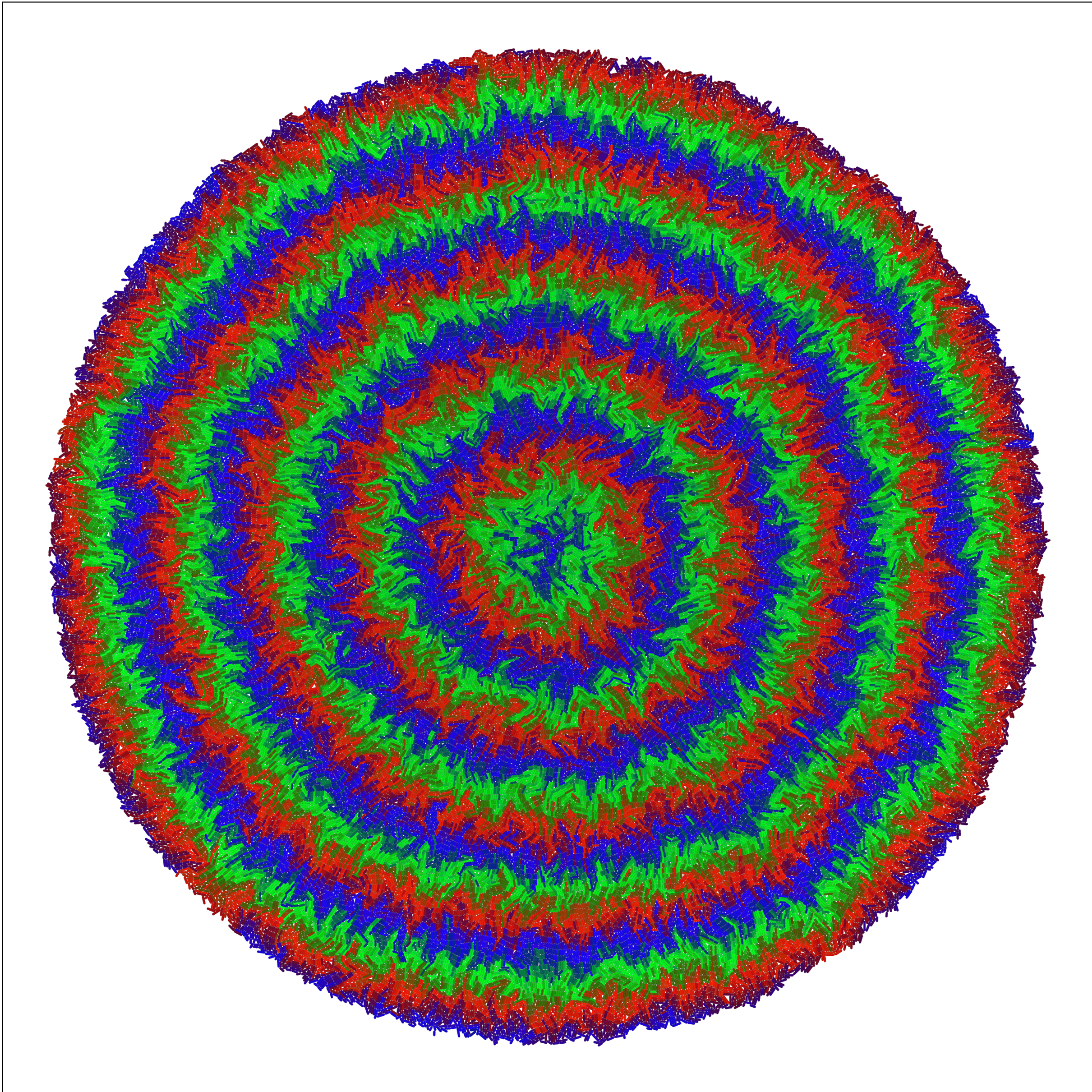

Supplement: Supplementary file 10 [file Image_1.TIF]
